# Relationship between Physical Performance and Frailty Syndrome in Older Adults: The Mediating Role of Physical Activity, Sedentary Time and Body Composition

**DOI:** 10.3390/ijerph18010203

**Published:** 2020-12-29

**Authors:** Irene Rodríguez-Gómez, Asier Mañas, José Losa-Reyna, Luis M. Alegre, Leocadio Rodríguez-Mañas, Francisco J. García-García, Ignacio Ara

**Affiliations:** 1 GENUD Toledo Research Group, Universidad de Castilla-La Mancha, 45071 Toledo, Spain; irene.rodriguez@uclm.es (I.R.-G.); Asier.Manas@uclm.es (A.M.); Jose.Losa@uclm.es (J.L.-R.); luis.alegre@uclm.es (L.M.A.); 2 CIBER of Frailty and Healthy Aging (CIBERFES), 28029 Madrid, Spain; leocadio.rodriguez@salud.madrid.org (L.R.-M.); franjogarcia@telefonica.net (F.J.G.-G.); 3 Geriatric Department, Hospital Virgen del Valle, 45071 Toledo, Spain; 4 Geriatric Department, Hospital Universitario de Getafe, 28905 Getafe, Spain

**Keywords:** movement behavior, sedentary time, muscle, fat mass, aging

## Abstract

The objectives were to clarify whether the relationship between physical performance and frailty was independently and jointly mediated by movement behaviors and body composition. We analyzed 871 older adults (476 women) from The Toledo Study for Healthy Aging. Skeletal muscle index (SMI) and fat index (FI) were determined using bone densitometry. Sedentary time (ST) and moderate-to-vigorous physical activity (MVPA) were assessed using accelerometry. The Frailty Trait Scale and The Short Physical Performance Battery (SPPB) were used to evaluate frailty and physical performance, respectively. Simple and multiple mediation analyses were carried out to determine the role of movement behaviors and body composition, adjusted for potential confounders. ST and MVPA acted independently as mediators in the relationship between SPPB and frailty (0.06% for ST and 16.89% for MVPA). FI also acted as an independent mediator in the same relationship (36.47%), while the mediation role of SMI was not significant. MVPA and FI both acted jointly as mediators in this previous relationship explaining 58.15% of the model. Our data support the fact that interventions should simultaneously encourage the promotion of MVPA and strategies to decrease the FI in order to prevent or treat frailty through physical performance improvement.

## 1. Introduction

It has been estimated that population aging will continue to increase until it rises to a value of 16% in the year 2050 [1]. For this reason, an important challenge in relation to health and social care resources is presented in order to decrease the risk of non-communicable diseases and disability [2]. One of the major problematic manifestations in older adults is frailty [3], which is acknowledged as a biological condition that produces a poor resolution of several physiological systems to maintain homoeostasis after a low-power stressor event [2,4]. In particular, this syndrome leads to loss of physical performance and dependence, which may negatively affect social and psychological abilities [5]. Physical frailty is linked to declines in multiple domains, including strength, balance, flexibility, reaction time, coordination and muscular and cardiovascular endurance [6]. Thus, preventing frailty and maximizing or maintaining functional independence in older people is a public health priority for seniors in an era of rapid population aging [7]. In order to alleviate these difficulties, increasing physical activity (PA) has been demonstrated to be an essential strategy to avoid the onset, progression, and perpetuation of this syndrome. For example, Mañas et al. (2019) found that achieving the physical activity guidelines relates to a more beneficial frailty profile [8], especially moderate-to-vigorous PA (MVPA), which predicts frailty in older adults [9]. Likewise, sedentary behavior patterns have been also associated with frailty levels [10]. Along the same lines as PA and lifestyle, lean mass decrease and fat mass increase have been widely linked with incident frailty in the elderly [11,12,13]. Moreover, the association between PA and movement behaviors and body composition has also been robustly proven and has been generally accepted for many years.

In spite of this evidence, the potential role of movement behaviors and body composition in the relationship between physical performance and frailty has not been studied yet. Mediation could be one of the roles that these predictors could play in the relation between variables like physical performance and frailty. Specifically, mediation analysis is a statistical method that can be used to elucidate the processes underlying an association between two variables and the extent to which the association can be modified, mediated, or confounded by a third variable [14].

Therefore, the first purpose of this study was to clarify whether the relationship between physical performance and frailty was independently mediated by movement behaviors and body composition. A secondary purpose was to identify whether mediators jointly explained the relationship between physical performance and frailty in the elderly to a greater extent.

## 2. Material and Methods

### 2.1. Study Design and Participants

This cross-sectional study used baseline data from The Toledo Study for Healthy Aging (TSHA). The methodology of the TSHA has been described elsewhere [15,16]. Briefly, the TSHA is a population prospective cohort study targeted at studying the determinants and consequences of frailty in community-dwelling individuals older than 65 years living in the province of Toledo, Spain. Data were collected in each of the waves in four stages. Firstly, computer-assisted face-to-face interviews with potential subjects were performed. Secondly, three nurses performed a physical examination and both clinical and performance tests at the participant’s home. Thirdly, for the blood sample, the subjects went to their health center in a fasted state. Fourthly, anthropometry data and body composition were obtained with dual energy X-ray absorptiometry (DXA). Finally, participants were asked to use an accelerometer for one week. Therefore, physical performance and frailty were obtained from the second stage and movement behaviors and body composition of the fourth. Particularly, related to movement behaviors, we differentiate between sedentary time (ST) and MVPA, while in relation to body composition, we did so between skeletal muscle index (SMI) and fat index (FI). Moreover, an extra model with the most relevant variables of both categories (movement behaviors and body composition) was carried out to study the mediation roles jointly.

Only the subjects who agreed to wear the accelerometer, to perform the DXA analysis, and who had their frailty status and physical performance determined were included in the present study (*n* = 871), since these tests comprised the inclusion criteria. Data were collected from July 2012 to June 2017. Signed informed consent was obtained from all participants. The study was approved by the Clinical Research Ethics Committee of the Toledo Hospital (approval code: 2010/93).

### 2.2. Procedures

#### 2.2.1. Frailty Status

To determine the frailty status, we used the Frailty Trait Scale (FTS) [17]. This scale is divided in 7 different dimensions: energy balance and nutrition, activity, nervous system, vascular system, weakness, endurance, and slowness. These domains become operational through 12 items. Each item score refers to a biological trait, which ranges from 0 (best score) to 4 (worst score), with the exception of the “chair test” that goes from 0 to 5 points. When appropriate, items are analyzed according to the quintile distribution of the item in the population.

The inclusion criteria for the participants were scoring at least 75% (9 of the 12) of the items included in this scale. The total FTS score ranged from 0 (less frailty) to 100 (more frailty).

#### 2.2.2. Movement Behaviors: Physical Activity and Sedentary Time

PA and ST were objectively measured by accelerometry (ActiTrainer and ActiGraph GT3X+; ActiGraph, LLC, Fort Walton Beach, FL, USA). The complete sample was invited to use an accelerometer, wearing it on their left hip during waking hours for 7 consecutive days, with the exception of bathing or swimming activities. Data using 60-s epochs were collected, and periods of at least 60 consecutive minutes of zero counts were established as non-wear time considering a range of two minutes of counts between zero and a hundred [18]. Older adult-specific cut-off points for vector magnitude counts per minute were used in this study. ST was defined with less than 200 counts per each minute [19] and MVPA was defined with 2751 or more counts per each minute [20]. Time spent in each of these movement behaviors was tallied per day and averaged over all available valid days. With regard to the exclusion criteria, the study excluded the results from participants with less than four valid days. A valid day was defined as at least 480 min (8 h).

#### 2.2.3. Body Composition: Lean and Fat Mass

All samples were evaluated using the same DXA instrument (Hologic QDR Discovery, Bedford, USA), which was calibrated using a lumbar spine phantom following the Hologic guidelines. To determine the whole and regional body composition, all the scans were performed with older people in the supine position, wearing light clothing and no metal, shoes or jewelry. The scans were analyzed using commercially available software (Physician’s Viewer; APEX System Software Version 3.1.2., Bedford, MA, USA). Furthermore, SMI was established from the ratio of the appendicular skeletal muscle mass and the height squared (kg·m^−2^) [21]. FI was calculated as the ratio of the whole body fat mass and the height squared (kg·m^−2^) [22].

#### 2.2.4. Physical Performance

For the assessment of physical performance in our study, the Short Physical Performance Battery (SPPB) was used [23]. This battery includes the following tests: (1) balance test: time holding 3 different positions (feet together, semi-tandem and full tandem) for a maximum of 10 s each; (2) usual gait speed: evaluated over a distance of three meters; and (3) chair stand test: time needed to get up and sit on the chair five times (arms crossed on the chest and as fast as possible). The test score was described in the original protocol [23].

#### 2.2.5. Anthropometrics and Confounding Variables

Subjects provided their age, sex and educational status. The educational levels were divided into three different categories: no studies, primary school, and secondary school or higher. In relation to the subject’s anthropometrics (height and body mass), a calibrated balance and stadiometer (Seca 220; Hamburg, Germany) was used following the following criteria: upright position, in underwear and barefoot. Additionally, height was assessed in the Frankfort plane. Body mass index (BMI) was calculated as body mass (kg) divided by height (m) squared (kg/m^2^).

### 2.3. Statistical Analysis

Analyses were carried out with the SPSS version 24.0 (IBM Corporation, SPSS, Inc., Chicago, IL, USA). The characteristics of the sample (mean and standard deviation) were determined through basic descriptive tests. Partial correlation coefficients were estimated to test the relationships between FTS, SPPB, movement behavior (ST and MVPA) and body composition variables (SMI and FI), controlling for age, sex, and educational status.

Different categories were created to determine the differences in FTS. For all the variables except SPPB, subjects were divided into three groups depending on the ST, MVPA, SMI and FI means as follows: low (1st quartile), medium (2nd and 3rd quartiles) and high (4th quartile). SPPB was divided into two groups because this classification has been previously associated with increased risk for loss of mobility and disability [24]: low functioning (9 score or less) and high functioning (10 score or more). These differences in FTS among the groups were established by means of different models according to covariance analyses (ANCOVA). The covariates used were different in each model: model 1 (age, sex and educational status), model 2 (model 1 + ST), model 3 (model 2 + MVPA), model 4 (model 3 + FI), model 5 (model 4 + SMI), and model 6 (model 5 + SPPB). Pairwise post-hoc hypotheses were tested using the Bonferroni correction for multiple comparisons.

A mediation analysis was performed to study whether the relationship between SPPB and FTS was mediated by movement behaviors (ST and MVPA) and body composition (FI and SMI). Two different approaches were used in the mediation analyses: simple and multiple mediation to cover the first and second objectives, respectively.

#### 2.3.1. Simple Mediation Analysis

The first regression model examined whether the association between SPPB and FTS was mediated by ST. The second regression model examined whether the association between SPPB and FTS was mediated by MVPA. The third regression model examined whether the association between SPPB and FTS was mediated by FI. The fourth regression model examined whether the association between SPPB and FTS was mediated by SMI. All regression models were adjusted for age, sex and educational status.

#### 2.3.2. Multiple Mediation Analysis

This was a more complex serial multiple mediator model that mixed PA and body composition mediators, specifying the sequence of mediation as follows: SPPB→MVPA→FI→FTS. MVPA and FI were selected against ST and SMI (respectively) because the correlations and the percentages of total effect mediated were higher in the first variables.

Both simple and multiple mediation models were estimated by the PROCESS macro for SPSS (IBM Corporation, SPSS, Chicago, IL, USA). The bootstrapping processes used were recommended by Preacher and Hayes [25] for testing mediation hypotheses, using a resampling procedure of 10,000 bootstrap samples. To ensure the statistical significance of the mediation effect, the Sobel test was also used. To estimate the serial mediation models, the order of variables had to have been previously determined.

In order to ensure the absence of multicollinearity problems, sensitivity analyses were conducted, removing the SPPB variables from the FTS. All analyses were performed in the complete sample and a significance level of *p* ≤ 0.05 was established in all models.

## 3. Results

Table 1 summarizes this study´s anthropometric and descriptive data (mean ± standard deviation). The final sample (*n* = 776) was decreased due to the exclusion criteria of the accelerometer wear time; in addition, some specific areas were excluded for the body composition analysis due to the existence of metal prosthesis or similar artefacts, which could affect the real results.

In order to test the relationships among FTS, SPPB, movement behavior variables and body composition variables adjusted for age, sex and educational status before conducting the mediation analysis, partial correlations were carried out (Table 2). SPPB was positively associated with MVPA (0.182), and negatively associated with FTS (−0.594), ST (−0.097) and FI (−0.169), but we did not detect any significant association between SPPB and SMI. FTS was positively associated with ST (0.190), FI (0.472) and SMI (0.246), and negatively associated with MVPA (−0.274).

**Figure 1:**
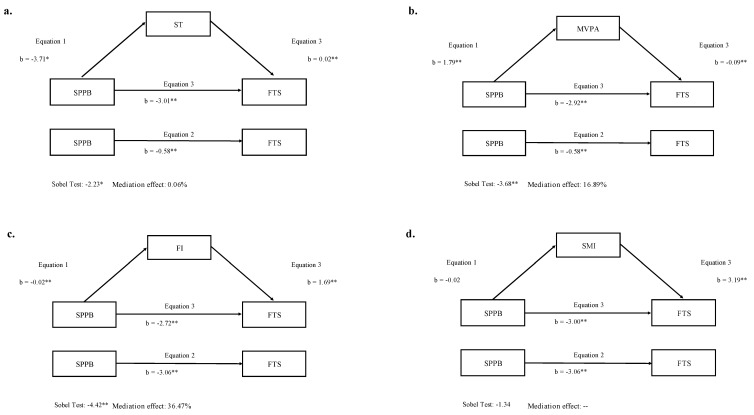
Simple mediation models of the associations between physical fitness (SPPB) and frailty (FTS), with movement behaviors and body composition as independent mediators in older adults. (**a**) Association between SPPB and FTS mediated by sedentary time (ST); (**b**) Association between SPPB and FTS mediated by moderate-to-vigorous physical activity (MVPA); (**c**) Association between SPPB and FTS mediated by fat index (FI); (**d**) Association between SPPB and FTS mediated by skeletal muscle index (SMI). * *p* < 0.05; ** *p* < 0.001. All models were adjusted for age, sex and educational status.

Simple mediation analysis diagrams are depicted in Figure 1. Overall, ST, MVPA and FI mediated the relationship between SPPB and FTS after controlling for age, sex and educational status. Related to Figure 1a, the relationship between SPPB and ST in the first regression equation turned out negative. In the second equation, the regression coefficient of SPPB on the dependent variable (FTS) was negative. In the third regression equation, the relationship between ST and FTS was positive, though between SPPB and FTS it remained negative when the mediator was incorporated in the regression model. In the same way, in Figure 1b,c, MVPA and FI acted as independent mediators of the relationship between SPPB and FTS. Although, the relationship between SPPB and MVPA was positive in the first regression equation and in the third regression equation, the relationship between MVPA and FTS was negative. Finally, both the indirect effect and the Sobel test were significant, confirming in these models the mediation role of ST (z = −2.23; *p* < 0.05), MVPA (z = −3.68; *p* < 0.01) and FI (z = −4.42; *p* < 0.01). The percentage of total effect mediated by ST was 0.06%, by MVPA was 16.89% and by FI was 36.47%. The mediation role was not significant for the SMI (z = −1.34; *p* > 0.05) (Figure 1d).

**Figure 2:**
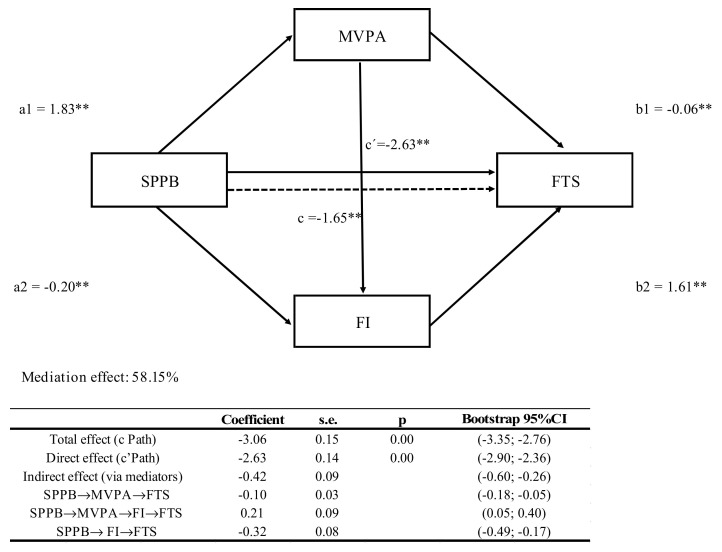
Multiple mediation model of the association between physical fitness (SPPB) and frailty (FTS) with moderate-to-vigorous physical activity (MVPA) and fat index (FI) as mediators in older adults. * *p* < 0.05; ** *p* < 0.001. Model was adjusted for age, sex and educational status.

The multiple mediation analysis diagram is depicted in Figure 2. In this model, MVPA and FI jointly mediated the relationship between SPPB and FTS after controlling for age, sex and educational status. All the paths in the model mediating this relationship between SPPB and FTS are shown in Figure 2. Thus, both the indirect effect and the Sobel test were significant (z = −3.17; *p* < 0.01 and z = −4.39; *p* < 0.01). This model explains 58.15% of the total variance.

When these analyses were re-run excluding SPPB variables from the FTS, similar results were obtained for both the simple and multiple mediations (Supplementary Files S1 and S2).

Supplementary File S3 shows group differences in FTS, according to the SPPB, ST, MVPA, FI and SMI categories. The high-functioning group had significantly lower FTS than the low functioning group for all sets of confounders in all models. In relation to movement behaviors, older people with greater ST had significantly higher FTS than those with less ST for the first five models. Older people that spent more time in MVPA obtained significantly less FTS than people who spent lower or medium time in this behavior for all models, except for the medium and high groups in model 6. In addition, we even found significant differences between the low and medium groups. When the sample was divided by FI, older people with more FI had significantly higher FTS than the people whose FI was low or medium for all the models, just like between the low and medium groups. Finally, older people with greater SMI showed significantly higher FTS than those with worse levels in the first three models.

## 4. Discussion

To the best of our knowledge, this investigation is the first to analyze the role of movement behaviors and body composition in the relationship between SPPB and FTS in older people using the simple and multiple mediation analyses. Our principal novel finding was that ST, MVPA and FI acted as independent mediators in the relationship between SPPB and FTS in this sample. Furthermore, MVPA and FI also acted together as mediators in the multiple model of the same relationship, explaining most of the variance. Therefore, these findings could provide a new pathway for trying to treat and prevent frailty.

### 4.1. Role of Movement Behaviors as Mediators

The simple mediation analysis confirmed the value of ST and MVPA as mediators, confirming the independent relationship between them and physical performance with frailty. Therefore, both movement behaviors seem to affect the relationships regarding frailty, as previous studies have also demonstrated [26,27]. In our study, older people with lower ST showed a significantly reduced frailty status than older people with higher or medium ST, while the opposite result was found in relation to the MVPA. Due to the fact that physical performance and frailty are outcomes closely related to successful aging [2], it would not be strange if both were related to common factors. There is a growing body of evidence showing a significant association between ST and frailty [28,29] and other health outcomes such as physical function or performance [2]. In the same way, there is also substantial evidence indicating that maintenance of an active lifestyle is central to successful aging, and the relationship between MVPA and frailty is now well established [2,8,30,31].

However, while the mediator effect of MVPA was of 16.9%, this effect was only 0.1% in the case of ST. Consequently, this study provides novel findings related to the higher importance of MVPA compared to ST in the associations concerning frailty and physical performance in this population group. Although, nowadays, more attention is paid to the relationship between ST and health variables, a recent study has indicated that MVPA, but not ST, predicts future frailty in older adults [9]. Similarly, a recent meta-analysis indicated that about 60−75 min/day of MVPA appeared to eliminate the increased risk of mortality associated with high sitting time among men and women [32]. In this way, not only benefits of MVPA are found, physically active older adults seem to have better physical performance and frailty profiles than those considered physically inactive, regardless of the ST [8]. Particularly, we found in our study that increasing physical performance in older people was linked to an increase in MVPA, which in fact could produce a decrease in frailty. Thus, MVPA as a mediator seems to be a potent preventive factor for frailty. In spite of the importance of the role of MVPA for reducing the risk of frailty progression through maintenance of physical performance, the effect of ST warrants further investigation.

### 4.2. Role of Body Composition as Mediator

In relation to body composition, the simple mediation analysis showed the mediator role of FI in the relationship between physical performance and frailty, while the mediation analysis showed no associations when SMI acted as mediator. Previous studies have already indicated that lean mass was not an important performance predictor for some frailty components [33]. Accordingly, authors such as Newman et al. (2006) have shown that muscle mass quantity, unlike quality, may not be a good predictor of mortality risk [34]. In this regard, Fougère et al. (2018) pointed out that the decline in lean mass is a component of the frailty syndrome but it not may be universally present [11]. Likewise, Ward et al. (2014) determined that adiposity, rather than lean mass, was the most influential body composition component negatively impacting physical-functional performance in community-dwelling older adults [35]. In fact, even in the presence of a high level of muscle mass, an individual with a disproportionately elevated level of adiposity (and hence body mass and low relative amount of muscle mass) may have detriments in performance [36]. Similarly, a recent prospective study also demonstrated the higher fat mass effect, which was associated with lower mortality in older frail women, while low lean mass was not a significant determinant of mortality [37]. In our study, we can also confirm these statements. In spite of the fact that FI and SMI increased the frailty status in older people and both were positively correlated with frailty; only FI was significantly correlated with the physical performance. Consequently, despite changes in body composition (lean and fat mass) in the elderly having a great impact on health status, functional capacity, quality of life and the progression of pathologies and disabilities [33,38], the importance of assessing the fat mass should be highlighted [33]. As a matter of fact, the relationship between fat mass and incident frailty has been widely demonstrated in the literature, both in cross-sectional [11,12,13] and longitudinal [12,39,40] studies. Most likely, the reason for the importance of FI could be that the greater amount of body fat, which leads to overloading by limiting movements, increases stress on joints and muscles and accentuates the risk of disability [41]. Furthermore, adipose tissue is an endocrine organ that secretes inflammatory and immune mediators that impact various metabolic functions [33], in addition to the fat infiltration in the muscle, which represents an overload for locomotion and reduces muscle quality and physical performance [42]. Therefore, when we used FI as the mediator variable, its effect was even higher than the effect of MVPA (36.5% vs. 16.9%), confirming the independent relationship between FI and physical performance with frailty. In consequence, the association between physical performance and frailty was widely established through the FI decrease.

### 4.3. Role of MVPA and FI as Multiple Mediators

The relevance of studying both mediators lies jointly in trying to explain the relationship between physical performance and frailty as much as possible. MVPA and FI extensively acted as independent mediators in this relationship against ST and SMI, respectively, given that the variance explained by these predictors was greater in both cases. Regular MVPA could be promoted not only as a way to prevent frailty and functional decline, but also as a way to achieve a healthier body composition or optimize physical performance at a given level of obesity [43]. Moreover, the evidence indicates that MVPA is a robust indicator of physical-function performance which depends on adiposity [35]. Therefore, it would be easy to consider a multiple mediation model that explains the role of MVPA and FI jointly as mediators. This model can be of help to clarify the relationships between both mediators and physical performance and frailty. In this way, our data show that both MVPA and FI were modified by physical performance and these changes could produce frailty variations. The effect of both mediators in the multiple model together were 58.2%; therefore, our findings suggest that it would be more appropriate to consider MVPA and FI jointly when the relationship between physical performance and frailty is studied in older people. Thus, to increase physical performance through MVPA increments and the reduction of fat mass seem to be an appropriate strategy to prevent frailty.

Frailty prevention is critical to maintaining independence and social interaction in the later years of life and is an important public health issue [44]. For this reason, it is highly relevant from a clinical perspective to identify the relationship between physical performance and frailty with movement behaviors and body composition (factors involved in the aging process).

Our study is not without limitations, causal inferences are restricted due to the cross-sectional nature of the study. Specific movement cut-off points for frail older adults would be essential to not underestimate their physical activity. Nonetheless, faced with this gap in the literature, we applied older adult-specific cut-off points for vector magnitude [19,20]. A key strength of our study is the fact that it adds more data about the relationship of frailty, an important contributor to the public health burden, with movement and body composition variables, helping towards an understanding of their mediator roles. Furthermore, the sample is constituted by a relatively large number of non-institutionalized older adults with objectively assessed frailty and physical performance, accelerometer derived movement behavior estimations and body composition determined through DXA.

## 5. Conclusions

In conclusion, this study demonstrates that MVPA and FI are the highest mediator factors in relation to the movement behaviors and body composition included in this study that assess the relationship between physical performance and frailty. In addition, this relationship is better explained if MVPA and FI are considered jointly as mediators in this population. From a practical perspective, our data support the fact that interventions should simultaneously encourage the promotion of MVPA and strategies to decrease the FI in order to prevent or treat frailty through physical performance improvement. Likewise, to increase MVPA rather than decrease ST would be a great strategy concerning frailty and physical performance. Future experimental research should go beyond this observational data and establish strategies that cause changes in physical activity and body composition in older people, especially in those with a more advanced frailty status.

## Figures and Tables

**Table 1:** Anthropometric and descriptive data.

Variables	Sample (*n* = 776)		
Sex (%)			
Men	360		46.4
Women	416		53.6
Age (years)	76.8	±	5.0
Body mass (kg)	73.5	±	12.7
Height (cm)	155.9	±	9.0
BMI (kg/m^2^)	30.3	±	4.8
Educational Level (%)			
No studies	492		63.9
Primary	169		21.9
Secondary	109		14.2
Body fat percentage (%)	36.6	±	7.7
Skeletal muscle index (kg/m^2^)	7.2	±	1.1
Fat index (kg/m^2^)	11.2	±	3.7
Accelerometer Wear Time (min/day)	799.3	±	82.3
Sedentary Time (min/d)	450.4	±	101.3
MVPA (min/d)	25.2	±	27.6
FTS (score)	38.0	±	14.4
SPPB (score)	8.4	±	3.2

Data are mean ± SD. BMI, body mass index; MVPA, moderate-to-vigorous physical activity; FTS, frailty trait scale; SPPB, short physical performance battery.

**Table 2:** Partial correlation coefficients among physical function, frailty, movement behaviors and body composition, corrected for age, sex and educational status.

	SPPB	FTS	ST	MVPA	FI	SMI
SPPB	-	−0.594 **	−0.097 *	0.182 **	−0.169 **	−0.050
FTS	-	-	0.190 **	−0.274 **	0.472 **	0.246 **
ST	-	-	-	−0.245 **	0.123 **	0.061
MVPA	-	-	-	-	−0.189 **	−0.084 *
FI	-	-	-	-	-	0.619 **
SMI	-	-	-	-	-	-

SPPB, short physical performance battery; FTS, frailty trait scale; ST, sedentary time; MVPA, moderate-to-vigorous physical activity; FI; fat index; SMI, skeletal muscle index. * *p* < 0.05, ** *p* < 0.01.

## Data Availability

There is an established infrastructure, including a website (http://www.ciberfes.es/) and a review committee, through which data requests are handled. The hospital reviews and determines the purposes for the data requests and what data can be released. Data requests can be sent to: Research and teaching unit, Virgen del Valle Hospital Ctra. Cobisa S/N, 45071 Toledo, Spain, info@estudiotoledo.com.

## Supplementary Materials

The following are available online at https://www.mdpi.com/1660-4601/18/1/203/s1, File S1: Simple mediation models of the associations between physical fitness (SPPB) and frailty (modified FTS), with movement behaviors and body composition as independent mediators in older adults; File S2: Multiple mediation model of the association between physical fitness (SPPB) and frailty (modified FTS), with moderate-to-vigorous physical activity (MVPA) and fat index (FI) as mediators in older adults; File S3: Frailty differences according to physical function (SPPB), sedentary time (ST), moderate-to-vigorous physical activity (MVPA), fat index (FI) and skeletal muscle index (SMI) categories.

## Abbreviations

PA	physical activity
MVPA	moderate-to-vigorous physical activity
TSHA	the Toledo study for healthy aging
DXA	dual energy X-ray absorptiometry
FTS	the frailty trait scale
ST	sedentary time
SMI	skeletal muscle index
FI	fat index
SPPB	the short physical performance battery (SPPB)

## References

[1] United Nations (2009). World Population Prospects: The 2008 Revision.

[2] Mañas A., Cruz B.D.P., García-García F.J., Guadalupe-Grau A., Ara I. (2017). Role of objectively measured sedentary behaviour in physical performance, frailty and mortality among older adults: A short systematic review. Eur. J. Sport Sci..

[3] Clegg A., Young J., Iliffe S., Rikkert M.O., Rockwood K. (2013). Frailty in elderly people. Lancet.

[4] Fried L.P., Tangen C.M., Walston J.D., Newman A.B., Hirsch C., Gottdiener J.S., Seeman T.E., Tracy R.P., Kop W.J., Burke G.L. (2001). Frailty in Older Adults: Evidence for a Phenotype. J. Gerontol. Ser. A Boil. Sci. Med. Sci..

[5] Chou C.-H., Hwang C.-L., Wu Y.-T. (2012). Effect of Exercise on Physical Function, Daily Living Activities, and Quality of Life in the Frail Older Adults: A Meta-Analysis. Arch. Phys. Med. Rehabil..

[6] Paw M.J.C.A., Chin A., van Uffelen J.G., Riphagen I., van Mechelen W. (2008). The functional effects of physical exercise training in frail older people. Sports Med..

[7] Lee W.J., Chen L.K., Peng L.N., Chiou S.T., Chou P. (2016). Personal mastery attenuates the adverse effect of frailty on declines in physical function of older people: A 6-year population-based cohort study. Medicine.

[8] Mañas A., Cruz B.D.P., Rodríguez-Gómez I., Leal-Martín J., Losa-Reyna J., Rodríguez-Mañas L., García-García F.J., Ara I. (2019). Dose-response association between physical activity and sedentary time categories on ageing biomarkers. BMC Geriatr..

[9] Mañas A., del Pozo-Cruz B., Rodríguez-Gómez I., Losa-Reyna J., Rodríguez-Mañas L., García-García F.J., Ara A. (2020). Which one came first: Movement behavior or frailty? A cross-lagged panel model in the Toledo Study for Healthy Aging. J. Cachexia Sarcopenia Muscle.

[10] del Pozo-Cruz B., Mañas A., Martín-García M., Marín-Puyalto J., García-García F.J., Rodriguez-Mañas L., Guadalupe-Grau A., Ara A. (2017). Frailty is associated with objectively assessed sedentary behaviour patterns in older adults: Evidence from the Toledo Study for Healthy Aging (TSHA). PLoS ONE.

[11] Fougère B., Sourdet S., Lilamand M., Tabue-Teguod M., Teysseyre B., Dupuy C., Vellas B., Rolland Y., Nourhashemi F., Van Kan G.A. (2018). Untangling the overlap between frailty and low lean mass: Data from Toulouse frailty day hospital. Arch. Gerontol. Geriatr..

[12] García-Esquinas E., García-García F.J., León-Muñoz L.M., Carnicero J.A., Guallar-Castillón P., Gonzalez-Colaço H.M., López-García E., Alonso-Bouzón C., Rodríguez-Mañas L., Rodríguez-Artalejo F. (2015). Obesity, fat distribution, and risk of frailty in two population-based cohorts of older adults in S pain. Obesity.

[13] Buch A., Carmeli E., Boker L.K., Marcus Y., Shefer G., Kis O., Berner Y., Stern N. (2016). Muscle function and fat content in relation to sarcopenia, obesity and frailty of old age—An overview. Exp. Gerontol..

[14] MacKinnon D.P. (2008). Introduction to Statistical Mediation Analysis.

[15] Carcaillon L., Blanco C., Alonso-Bouzón C., Alfaro-Acha A., Garcia-García F.-J., Rodríguez-Mañas L. (2012). Sex Differences in the Association between Serum Levels of Testosterone and Frailty in an Elderly Population: The Toledo Study for Healthy Aging. PLoS ONE.

[16] García-García F.J., Avila G.G., Alfaro-Acha A., Andres M.S.A., Lanza M.D.L.A.D.L.T., Aparicio M.V.E., Aparicio S.H., Zugasti J.L.L., Reus M.G.-S., Rodriguez-Artalejo F. (2011). The prevalence of frailty syndrome in an older population from Spain. The Toledo study for healthy aging. J. Nutr. Health Aging.

[17] García-García F.J., Carcaillon L., Fernandez-Tresguerres J., Alfaro A., Larrion J.L., Castillo C., Rodríguez-Mañas L. (2014). A New Operational Definition of Frailty: The Frailty Trait Scale. J. Am. Med. Dir. Assoc..

[18] Colley R., Gorber S.C., Tremblay M.S. (2010). Quality control and data reduction procedures for accelerometry-derived measures of physical activity. Health Rep..

[19] Aguilar-Farías N., Brown W.J., Peeters G. (2014). (Geeske) ActiGraph GT3X+ cut-points for identifying sedentary behaviour in older adults in free-living environments. J. Sci. Med. Sport.

[20] Santos-Lozano A.S., Santín-Medeiros F., Cardon G., Torres-Luque G., Bailón R., Bergmeir C., Ruiz J.R., Lucia A., Garatachea N. (2013). Actigraph GT3X: Validation and Determination of Physical Activity Intensity Cut Points. Int. J. Sports Med..

[21] Cruz-Jentoft A.J., Baeyens J.P., Bauer J.M., Boirie Y., Cederholm T., Landi F., Martin F.C., Michel J.-P., Rolland Y., Schneider S.M. (2010). Sarcopenia: European consensus on definition and diagnosisReport of the European Working Group on Sarcopenia in Older People. Age Ageing.

[22] Alcazar J., Rodriguez-Lopez C., Ara I., Alfaro-Acha A., Rodríguez-Gómez I., Navarro-Cruz R., Losa-Reyna J., García-García F.J., Alegre L.M. (2018). Force-velocity profiling in older adults: An adequate tool for the management of functional trajectories with aging. Exp. Gerontol..

[23] Guralnik J.M., Simonsick E.M., Ferrucci L., Glynn R.J., Berkman L.F., Blazer D.G., Scherr P.A., Wallace R.B. (1994). A Short Physical Performance Battery Assessing Lower Extremity Function: Association With Self-Reported Disability and Prediction of Mortality and Nursing Home Admission. J. Gerontol..

[24] Guralnik J.M., Ferrucci L., Pieper C.F., Leveille S.G., Markides K.S., Ostir G.V., Studenski S., Berkman L.F., Wallace R.B. (2000). Lower Extremity Function and Subsequent Disability: Consistency Across Studies, Predictive Models, and Value of Gait Speed Alone Compared With the Short Physical Performance Battery. J. Gerontol. Ser. A Boil. Sci. Med. Sci..

[25] Preacher K.J., Hayes A.F. (2008). Asymptotic and resampling strategies for assessing and comparing indirect effects in multiple mediator models. Behav. Res. Methods.

[26] Rodríguez-Gómez I., Rodríguez-Mañas L., Losa-Reyna J., Rodríguez-Mañas L., Chastin S.F., Alegre L.M., García-García F.J., Ara I. (2020). Prospective Changes in the Distribution of Movement Behaviors Are Associated With Bone Health in the Elderly According to Variations in their Frailty Levels. J. Bone Miner. Res..

[27] Rodríguez-Gómez I., Ara I., Losa-Reyna J., Rodríguez-Mañas L., Chastin S., Alegre L.M., García-García F.J., Ara I. (2019). The Impact of Movement Behaviors on Bone Health in Elderly with Adequate Nutritional Status: Compositional Data Analysis Depending on the Frailty Status. Nutrition.

[28] Coqueiro R., De Queiroz B.M., Oliveira D.S., Das Merces M.C., Carneiro J.A., Pereira R., Fernandes M.H. (2016). Cross-sectional relationships between sedentary behavior and frailty in older adults. J. Sports Med. Phys. Fit..

[29] Schwenk M., Mohler J., Wendel C., D’Huyvetter K., Fain M., Taylor-Piliae R., Najafi B. (2015). Wearable Sensor-Based In-Home Assessment of Gait, Balance, and Physical Activity for Discrimination of Frailty Status: Baseline Results of the Arizona Frailty Cohort Study. Gerontology.

[30] Blodgett J.M., Theou O., Kirkland S., Andreou P., Rockwood P.K. (2015). The association between sedentary behaviour, moderate–vigorous physical activity and frailty in NHANES cohorts. Maturitas.

[31] Marques E.A., Baptista F., Santos D.A., Silva A.M., Mota J., Sardinha L.B. (2014). Risk for losing physical independence in older adults: The role of sedentary time, light, and moderate to vigorous physical activity. Maturitas.

[32] Ekelund U., Steene-Johannessen J., Brown W.J., Fagerland M.W., Owen N., Powell K.E., Bauman A., Lee I.-M. (2016). Does physical activity attenuate, or even eliminate, the detrimental association of sitting time with mortality? A harmonised meta-analysis of data from more than 1 million men and women. Lancet.

[33] Falsarella G.R., Gasparotto L.P.R., Barcelos C.C., Moretto M.C., Pascoa M.A., Ferreira T.C.B.R., Coimbra A.M.V., Coimbra I.B. (2015). Body composition as a frailty marker for the elderly community. Clin. Interv. Aging.

[34] Newman A.B., Kupelian V., Visser M., Simonsick E.M., Goodpaster B.H., Kritchevsky S.B., Tylavsky F.A., Rubin S.M., Harris T.B. (2006). Strength, But Not Muscle Mass, Is Associated With Mortality in the Health, Aging and Body Composition Study Cohort. J. Gerontol. Ser. A Boil. Sci. Med. Sci..

[35] Ward C.L., Valentine R.J., Evans E.M. (2014). Greater Effect of Adiposity Than Physical Activity or Lean Mass on Physical Function in Community-Dwelling Older Adults. J. Aging Phys. Act..

[36] Valentine R.J., Misic M.M., Rosengren K.S., Woods J.A., Evans E.M. (2009). Sex impacts the relation between body composition and physical function in older adults. Menopause.

[37] Zaslavsky O., Rillamas-Sun E., Li W., Going S., Datta M., Snetselaar L., Zelber-Sagi S. (2016). Association of dynamics in lean and fat mass measures with mortality in frail older women. J. Nutr. Health Aging.

[38] Fantin F., Di Francesco V., Fontana G., Zivelonghi A., Bissoli L., Zoico E., Rossi A., Micciolo R., Bosello O., Zamboni M. (2007). Longitudinal Body Composition Changes in Old Men and Women: Interrelationships With Worsening Disability. J. Gerontol. Ser. A Boil. Sci. Med. Sci..

[39] Fugate Woods N., LaCroix A.Z., Gray S.L., Aragaki A., Cochrane B.B., Brunner R.L., Masaki K., Murray A., Newman A.B. (2005). Frailty: Emergence and consequences in women aged 65 and older in the Women’s Health Initiative Observational Study. J. Am. Geriatr. Soc..

[40] Barzilay J.I., Blaum C., Moore T., Xue Q.L., Hirsch C.H., Walston J.D., Fried L.P. (2007). Insulin resistance and inflammation as precursors of frailty: The Cardiovascular Health Study. Arch. Intern. Med..

[41] Santos W.T.D., Rodrigues E.d.C., Mainenti M.R.M. (2014). Muscle performance, body fat, pain and function in the elderly with arthritis. Acta Ortop. Bras..

[42] Waters D.L., Baumgartner R.N. (2011). Sarcopenia and Obesity. Clin. Geriatr. Med..

[43] Castaneda-Gameros D., Redwood S., Thompson J.L. (2018). Physical Activity, Sedentary Time, and Frailty in Older Migrant Women From Ethnically Diverse Backgrounds: A Mixed-Methods Study. J. Aging Phys. Act..

[44] Guralnik J.M., Lacroix A.Z., Abbott R.D., Berkman L.F., Satterfield S., Evans D.A., Wallace R.B. (1993). Maintaining Mobility in Late Life. I. Demographic Characteristics and Chronic Conditions. Am. J. Epidemiol..

