# Supplementary material for: Relationship between Physical Performance and Frailty Syndrome in Older Adults: The Mediating Role of Physical Activity, Sedentary Time and Body Composition
Source: Int J Environ Res Public Health. 2020 Dec 29;18(1):203. doi: 10.3390/ijerph18010203 (PMC7794741; doi:10.3390/ijerph18010203)
Supplement: Supplementary file 1 [file ijerph-18-00203-s001.pdf]

**Supplementary File 1**. Simple mediation models of the associations between physical fitness (SPPB) and frailty (modified FTS), with movement behaviors and body composition as independent mediators in older adults.

a. Association between SPPB and modified FTS mediated by sedentary time (ST);

b. Association between SPPB and modified FTS mediated by moderate-to-vigorous physical activity (MVPA);

c. Association between SPPB and modified FTS mediated by fat index (FI);

d. Association between SPPB and modified FTS mediated by skeletal muscle index (SMI). *p<0.05; **p<0.001. FTS*: modified FTS with variables included in the SPPB removed. All models were adjusted for age, sex and educational status.

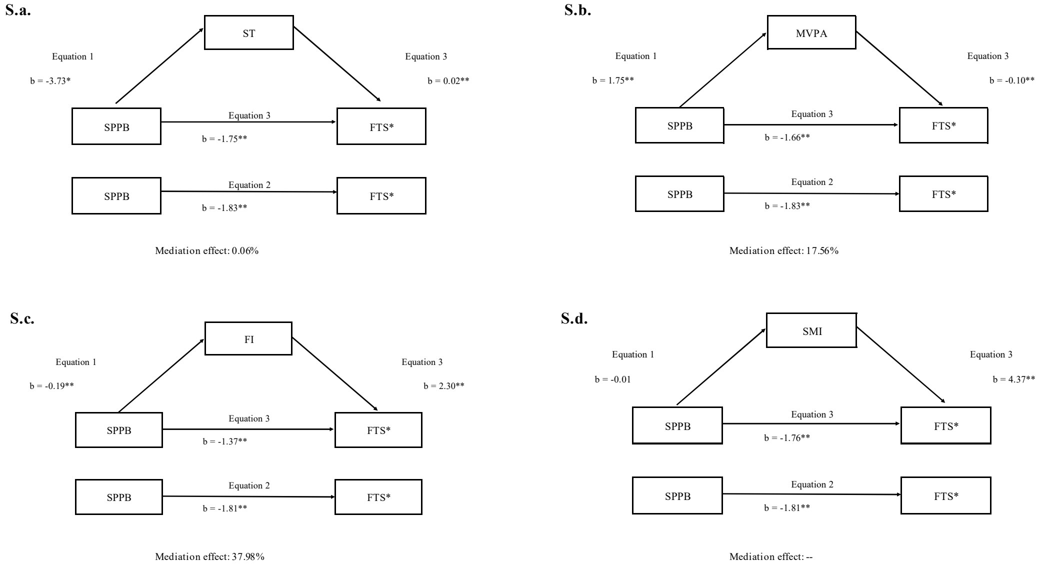

**Supplementary File 2.** Multiple mediation model of the association between physical fitness (SPPB) and frailty (modified FTS), with moderate-to-vigorous physical activity (MVPA) and fat index (FI) as mediators in older adults.

*p<0.05; **p<0.001. FTS*: modified FTS with variables included in the SPPB removed. Model was adjusted for age, sex and educational

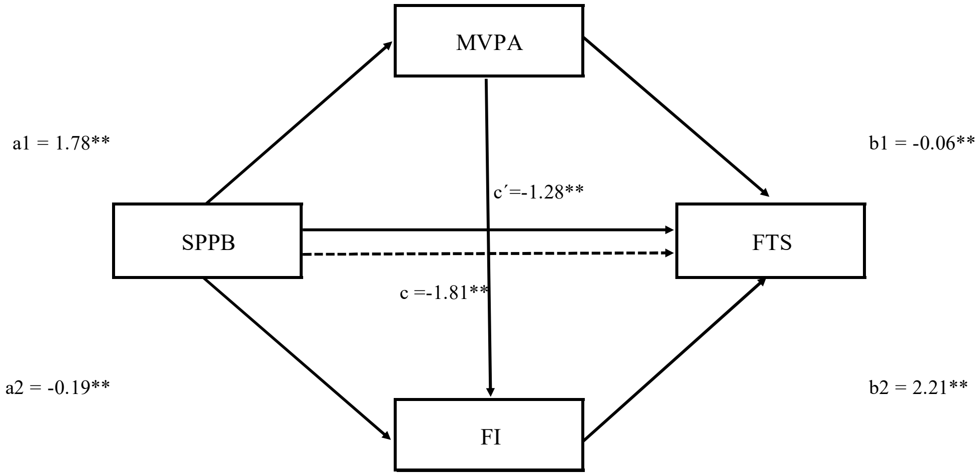


Mediation effect: 40.41%

| | Coefficient | s.e. | p | Bootstrap 95%CI |
|---|---|---|---|---|
| Total effect (c Path) | -1,81 | 0,18 | 0,00 | (-2.17; -1.46) |
| Direct effect (c'Path) | -1,28 | 0,16 | 0,00 | (-1.59; -0.97) |
| Indirect effect (via mediators) | -0,53 | 0,11 | | (-0.76; -0.33) |
| SPPB→MVPA→FTS | -0,11 | 0,04 | | (-0.20; -0.05) |
| SPPB→MVPA→FI→FTS | 0,31 | 0,11 | | (0.10; 0.53) |
| SPPB→ FI→FTS | -0,42 | 0,1 | | (-0.63; -0.23) |

**Supplemental File 3** There is an established infrastructure, including a website (http://http://www.ciberfes.es/) and a review committee, through which data requests are handled. The hospital reviews and determines the purposes for the data requests and what data can be released. Data requests can be sent to: Research and teaching unit, Virgen del Valle Hospital Ctra. Cobisa S/N, 45071 Toledo – Spain, info@estudiotoledo.com.

**.** Frailty differences according to physical function (SPPB), sedentary time (ST), moderate-to-vigorous physical activity (MVPA), fat index (FI) and skeletal muscle index (SMI) categories.

| Model | SPPB | | ST | | | MVPA | | |
|---|---|---|---|---|---|---|---|---|
| | Low (n=412) | High (n=332) | Low (n=194) | Medium (n=388) | High (n=194) | Low (n=194) | Medium (n=388) | High (n=194) |
| 1 | 43.9 ± 0.6 | 30.4 ± 0.7* | 35.1 ± 1.0 | 37.3 ± 0.7 | 42.2 ± 1.0*† | 43.9 ± 1.0 | 37.9 ± 0.7 | 32.6 ± 1.0*†ϴ |
| 2 | 43.7 ± 0.6 | 30.5 ± 0.7* | - | - | - | 43.1 ± 1.0 | 38.0 ± 0.7 | 33.0 ± 1.0*†ϴ |
| 3 | 43.2 ± 0.6 | 31.2 ± 0.7* | 35.9 ± 1.0 | 37.4 ± 0.7 | 41.2 ± 1.0*† | - | - | - |
| 4 | 42.8 ± 0.5 | 31.6 ± 0.6* | 37.2 ± 0.9 | 37.0 ± 0.6 | 40.5 ± 0.9*† | 42.2 ± 0.9 | 37.6 ± 0.6 | 34.0 ± 0.9*†ϴ |
| 5 | 42.8 ± 0.5 | 31.6 ± 0.6* | 37.2 ± 0.9 | 37.1 ± 0.6 | 40.5 ± 0.9*† | 42.2 ± 0.9 | 37.6 ± 0.6 | 34.0 ± 0.9*†ϴ |
| 6 | - | - | 37.0 ± 0.7 | 37.5 ± 0.5 | 39.3 ± 0.7 | 40.4 ± 0.8 | 37.4 ± 0.5 | 36.0 ± 0.7*ϴ |

| Model | FI | | | SMI | | |
|---|---|---|---|---|---|---|
| | Low (n=193) | Medium (n=388) | High (n=193) | Low (n=193) | Medium (n=388) | High (n=193) |
| 1 | 28.3 ± 1.0 | 37.5 ± 0.6 | 48.6 ± 1.0*†ϴ | 32.3 ± 1.1 | 38.0 ± 0.7 | 43.1 ± 1.0*†ϴ |
| 2 | 28.7 ± 1.0 | 37.5 ± 0.6 | 48.2 ± 1.0*†ϴ | 32.5 ± 1.0 | 38.1 ± 0.7 | 42.8 ± 1.0*†ϴ |
| 3 | 29.5 ± 1.0 | 37.3 ± 0.6 | 47.9 ± 1.0*†ϴ | 32.7 ± 1.0 | 38.3 ± 0.6 | 42.3 ± 1.0*†ϴ |
| 4 | - | - | - | 38.0 ± 1.0 | 37.8 ± 0.6 | 38.0 ± 1.0 |
| 5 | 29.7 ± 1.0 | 37.3 ± 0.6 | 47.5 ± 1.1*†ϴ | - | - | - |
| 6 | 30.9 ± 0.8 | 37.6 ± 0.5 | 45.5 ± 0.9*†ϴ | 37.5 ± 0.9 | 37.5 ± 0.5 | 38.7 ± 0.8 |

Data are ± SEM.

Covariates for each model: Model 1 (age, sex and educational status); Model 2 (Model 1+ ST); Model 3 (Model 2 + MVPA); Model 4 (Model 3 + FI); Model 5 (Model 4 + SMI); Model 6 (Model 5 + SPPB).

* $p$<0.05 for High group vs. Low group. † $p$<0.05 for High group vs. Medium group. ϴ $p$<0.05 for Medium group vs. Low group.
